# Age of acquisition of disease-causal human papillomavirus infection to high-grade cervical intraepithelial neoplasia (CIN2+) in England

**DOI:** 10.1186/s12879-026-12686-z

**Published:** 2026-03-31

**Authors:** Alhaji Cherif, Kayla Engelbrecht, Olga Ovcinnikova-Hutchings, Dionysios Ntais, Xuedan You

**Affiliations:** 1 Biostatistics and Research Decision Sciences (BARDS), Merck & Co., Inc., 2025 E Scott Ave, Rahway, NJ USA; 2 Value, Access and Devolved Nations (VAD), MSD (UK) Limited, London, UK; 3 Value and Implementation (V&I), Merck & Co., Inc., 2025 E Scott Ave, Rahway, NJ USA

**Keywords:** Human papillomavirus, England, Discrete event model, Cervical intraepithelial neoplasia, Cervical cancer, Disease-causal HPV infection, Age distribution, Adult, Vaccination

## Abstract

**Background:**

The human papillomavirus (HPV) is the primary causative risk factor for developing cervical intraepithelial neoplasia (CIN), which can progress to cervical cancer in women. The age at which women in England acquire a causal HPV infection that progresses into CIN and subsequently cervical cancer is not well understood. HPV immunisation was introduced in England in 2008 and catch-up vaccination is available up to the age of 25.

**Methods:**

A published discrete event model was modified to estimate the median age of acquisition of causal HPV infection for CIN2+ (CIN2, CIN2/3, CIN3, and adenocarcinoma in situ) among women diagnosed with CIN2 + in England. The model simulates 1,000 women progressing through causal HPV infection, CIN2 + onset, and CIN2 + diagnosis to determine the optimal time delay between causal infection and CIN2 + diagnosis by comparing the predicted age distribution of CIN2 + incidence to real-world diagnosis data. Scenario analyses were conducted to test the impact of uncertainty on the model results by implementing alternate parameterisations for the time from causal HPV infection to CIN2 + onset. Two sensitivity analyses were conducted; the first assumed the time from causal infection to onset of CIN2 + was age-dependent, and the second used an alternate parametric exponential distribution in the model.

**Results:**

The model predicted that the median ages of causal infection and disease diagnosis (95% confidence interval [CI]) were 26.43 (26.36, 26.50) and 32.42 (32.37, 32.47) years, respectively, based on an offset (defined as the time between causal HPV infection and CIN2 + diagnosis) of 6.17 (6.08, 6.26) years. A substantial proportion of causal HPV infections occurred after the age of 25 (56.24%, 95% CI 55.93, 56.55). The model results were robust to variations in inputs and parameterisation tested in scenario and sensitivity analyses.

**Conclusions:**

There is a substantial burden of disease-causal HPV infection and high-grade cervical disease among women in England over the age of 25 years. Vaccinating women before acquiring disease-causal HPV infection can prevent progression to CIN2+, and subsequent cervical cancer. This supports the need for continued vaccination catch-up opportunities, in addition to potential benefits of HPV vaccination strategies beyond the eligible age within the national immunisation programme.

**Clinical trial number:**

Not applicable.

**Supplementary Information:**

The online version contains supplementary material available at 10.1186/s12879-026-12686-z.

## Background

Human papillomavirus (HPV) is a common sexually transmitted infection and the primary causative risk factor for cervical intraepithelial neoplasia (CIN) and cervical cancer [1]. Between January 2006 and June 2020, 335,228 women aged 20–64 years were diagnosed with CIN3, and 29,968 women were diagnosed with cervical cancer in England [2]. The risk of acquiring HPV infections peaks in adolescence and young adulthood soon after sexual debut and declines with age in most women [3, 4]. Although most HPV infections clear within a few years [5], women remain at risk of new HPV infections, including persistent infection, through adulthood [6–8], which can be disease causal for CIN and cervical cancer [8].

HPV vaccination is highly effective in preventing HPV infection and CIN grades 2, 2/3, 3, and adenocarcinoma in situ (CIN2+) [9–11], and screening remains an effective secondary prevention strategy. In 2008, a school-based national immunisation programme (NIP) was introduced in the United Kingdom (UK) to adolescent girls, and currently, catch-up vaccination is available up until the age of 25 [12]. Further, the National Health Service Cervical Screening Programme (NHS CSP) in England is currently offered to women and people with a cervix aged 25 to 64 years, with invitations every 3 years (if aged 25 to 49 years) or 5 years (if aged 50 to 64 years) [13]. Clinical studies demonstrate the safety and efficacy of HPV vaccination in women aged 16–26 years [14, 15] and in women up to 45 years [16–20] against new infection and reinfection. Efficacy and immunogenicity data for the HPV vaccine in women aged 26–45 years support that catch-up vaccination may still provide benefit for women who were not previously vaccinated as adolescents or young adults [17, 19–22].

Previous studies have examined the natural history of HPV infections and CIN lesions across different age groups. The risk of progression of HPV infection to CIN2 + was similar in women over 25 years of age and in women aged 15–25 years in the control arms of the VIVIANE and PATRICIA studies, respectively [23, 24]. Previous modelling studies have projected the median age of HPV infection in women (not necessarily a causal infection) [25] and the median age of causal HPV infection leading to CIN2+ [26] and resulting in cervical cancer [27] in women in the United States (US). While analyses of control arms of phase 3 studies [23, 24] and US modelling studies [25–27] add to the evidence base toward a better understanding of the natural history of HPV infection, no previous study has examined the age of acquisition of causal HPV infection that results in CIN2 + in women in England. An understanding of the age of disease-causal HPV infection can help inform HPV vaccination policies aimed at alleviating the burden of HPV infection and cervical precancer and cancer. The objective of this study was to estimate the age distribution of disease-causal HPV infection in women diagnosed with CIN2 + in England.

## Methods

### Model overview and structure

**Fig. 1:**
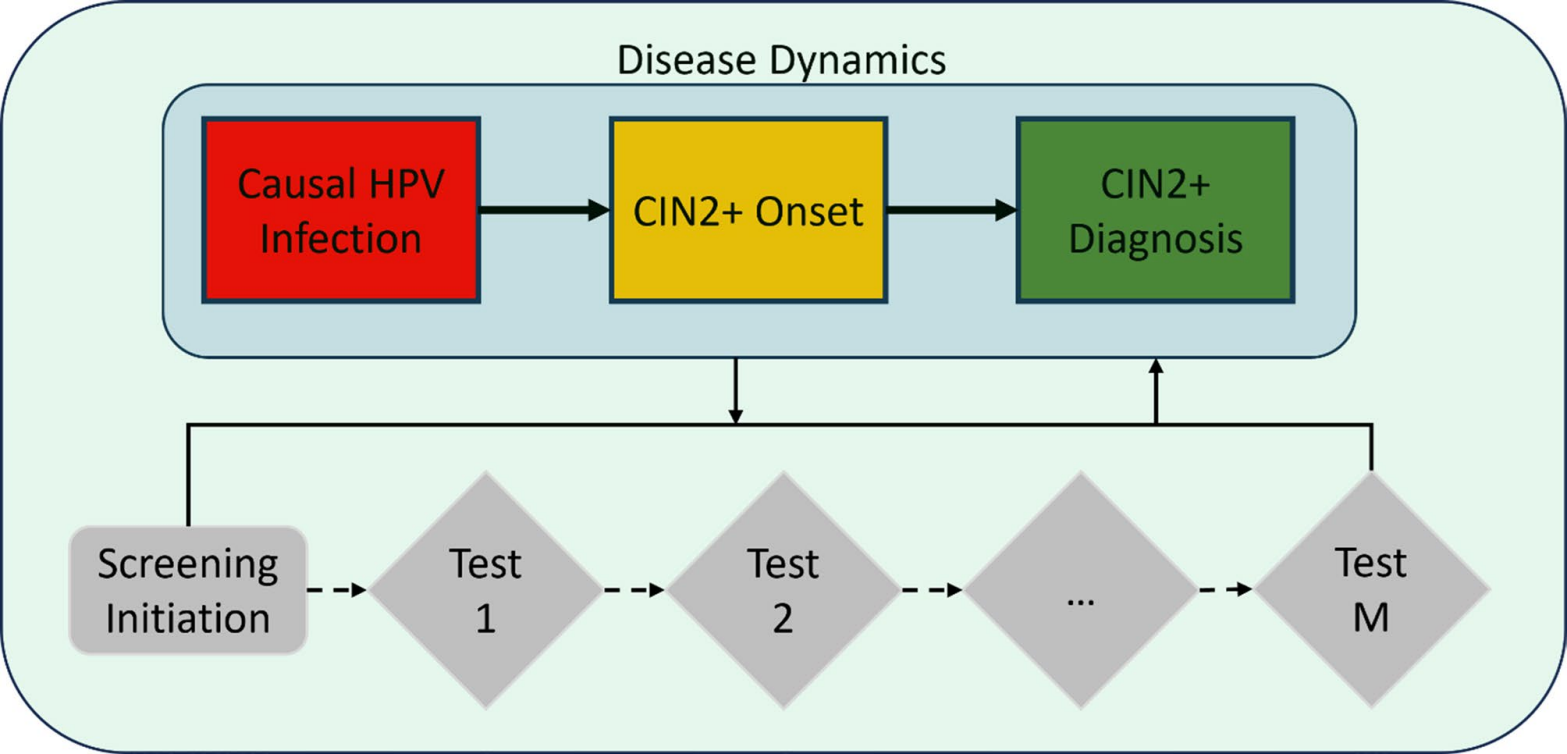
Discrete event simulation model structure. CIN2+, cervical intraepithelial neoplasia grades 2, 2/3, 3, and adenocarcinoma; HPV, human papillomavirus

A published discrete event model [26] was modified to estimate the age of entering three health states: causal HPV infection, CIN2 + onset, and CIN2 + diagnosis based on screening in England (Fig. 1). CIN2 + was defined was CIN2, CIN2/3, CIN3, and adenocarcinoma in situ. Invasive cancers (squamous cell carcinoma and invasive adenocarcinoma) are not included in the study outcomes. The model simulates 1,000 women who may be diagnosed with CIN2 + during their lifetime in England. Women enter the model at causal HPV infection onset or at age 25 (start of screening), whichever occurs earlier. The model estimates the age at causal HPV infection onset by assuming that the unknown shape of the cumulative age distribution is similar to the observed age distribution of CIN2 + diagnosis, except that it is shifted earlier by a fixed “offset”. This offset represents the time from HPV infection to CIN2 + diagnosis. Women with causal HPV infection can progress to CIN2 + onset (undiagnosed), and women with CIN2 + onset can then be diagnosed through screening. Using a gamma distribution, the model predicts the CIN2 + age distribution, and the offset time is varied to get a best fit of the predicted CIN2 + age distribution to the observed distribution. From the best fit, the model derives the age distribution (and median value) of the age at causal infection. We report the average of 100 replicates of the model for the base case analysis and 10 replicates for the other analyses (e.g., sensitivity and scenario analyses).

### Model inputs

Supplemental Table 1 summarizes the input values for each parameter in the model. The model does not incorporate the impact of HPV vaccination, which was introduced in 2008 to girls aged 12–13 years and with a catch-up programme for females aged 14–18 years in 2008–2010 [12]. Therefore, real-world data for CIN2 + diagnosis and cervical cancer screening were sourced from the 2007–2008 NHS CSP in England. During this time, the most common screening method was liquid-based cytology, which has an estimated sensitivity of approximately 80.1% [28]. In the base case, we assumed a gamma distribution (α = 1 and β = 1) for the time from causal infection to CIN2 + onset after a fixed 6-month minimum time to onset. This distribution is based on data on the time from persistent infection to onset of CIN2 + from the VIVIANE bivalent HPV (2vHPV) vaccine clinical trials [24] and the FUTURE I quadrivalent HPV (4vHPV) vaccine clinical trials [5]; this is also the distribution that was used by Prabhu et al. (2021) [26].

#### Age of CIN2 + diagnosis

Data from the NHS CSP in England from 2007 to 2008 were used to derive the incidence of CIN2 + for each age group [29]. Approximately 8% of the CIN2 + cases occurred before the earliest age of screening (25 years of age). Since the model requires screening to diagnose CIN2+, cases diagnosed at < 25 years were excluded from the model calibration. The model was adjusted to incorporate the excluded 8% of the females when reporting results (i.e., medians, confidence intervals [CIs], and graphs) for the base case and scenario analyses.

#### Age of screening

Three-year screening rates were used to estimate the age of females at subsequent screens in England, which was obtained from the NHS CSP in England for 2007–2008 [29]. Annual screening rates were calculated using the 3-year screening rates by the following equation: Annual rate = 1-{1 - [3 year screening rate]} ^ (1/3).

### Model assumptions

Our model assumes that the shape of the distribution for the age of causal HPV infection is similar to the age distribution for CIN2 + diagnosis and does not vary according to HPV genotype. The model also assumes that CIN2 + detection can only occur by screening tests for the ages during which screening takes place. The time to the next screening event is independent of how frequently the simulated female has been screened. Differences in screening frequency among risk groups or sexual behaviours were not explicitly incorporated into the model.

### Scenario analyses

A series of scenario analyses were performed in which the alpha and beta parameters of the gamma distribution were varied to evaluate the sensitivity of model results to variations in input parameters. Alternative gamma distributions were derived utilising data from the FUTURE I clinical trial [5] and by an assumption regarding the extent of censoring of CIN2 + cases after 3 years. We calculated the cumulative percentage of CIN2 + cases observed after 1, 2, and 3 years (58%, 83%, and 100%, respectively). However, not all of the persistent infections or CIN1 had cleared or progressed to CIN2 + by 3 years, and these females remained at risk of progression to CIN2 + and were considered “censored”. Because it is unknown what percentage of these females might progress to CIN2+, we assumed 0%, 5%, 10%, or 20% of these females were censored and would eventually progress to CIN2+. Under each censoring assumption, we recalculated the cumulative percentage of total CIN2 + cases observed over the first 3 years and refitted the gamma distribution so that the model time from infection to onset (i.e., the offset) was varied one at a time around their base case values for the following scenarios using a gamma distribution: (1) 0% censoring + gamma (0.7, 1.1); (2) 5% censoring + gamma (0.5, 2.0); (3) 10% censoring + gamma (0.5, 2.75); and (4) 20% censoring + gamma (0.5, 4.0). Data were statistically analysed using analysis of variance after verification of data normality and homogeneity of variances.

### Sensitivity analyses

A sensitivity analysis was conducted using an alternate parametric exponential distribution with a lambda of 1.2 for simulating the time from causal infection to onset of CIN2+. In the base case, we assume the time from causal infection to CIN2 + onset does not depend on age. However, it is hypothesised that the time from causal infection to CIN2 + onset increases with the age at which the causal infection is acquired (i.e., infections acquired at older ages progress more slowly). Therefore, we also included a sensitivity analysis that examined the impact of age dependence in the time from causal infection to CIN2 + onset when estimating the median age of causal infection (see Appendix 1 for more details).

## Results

### Base case

**Fig. 2:**
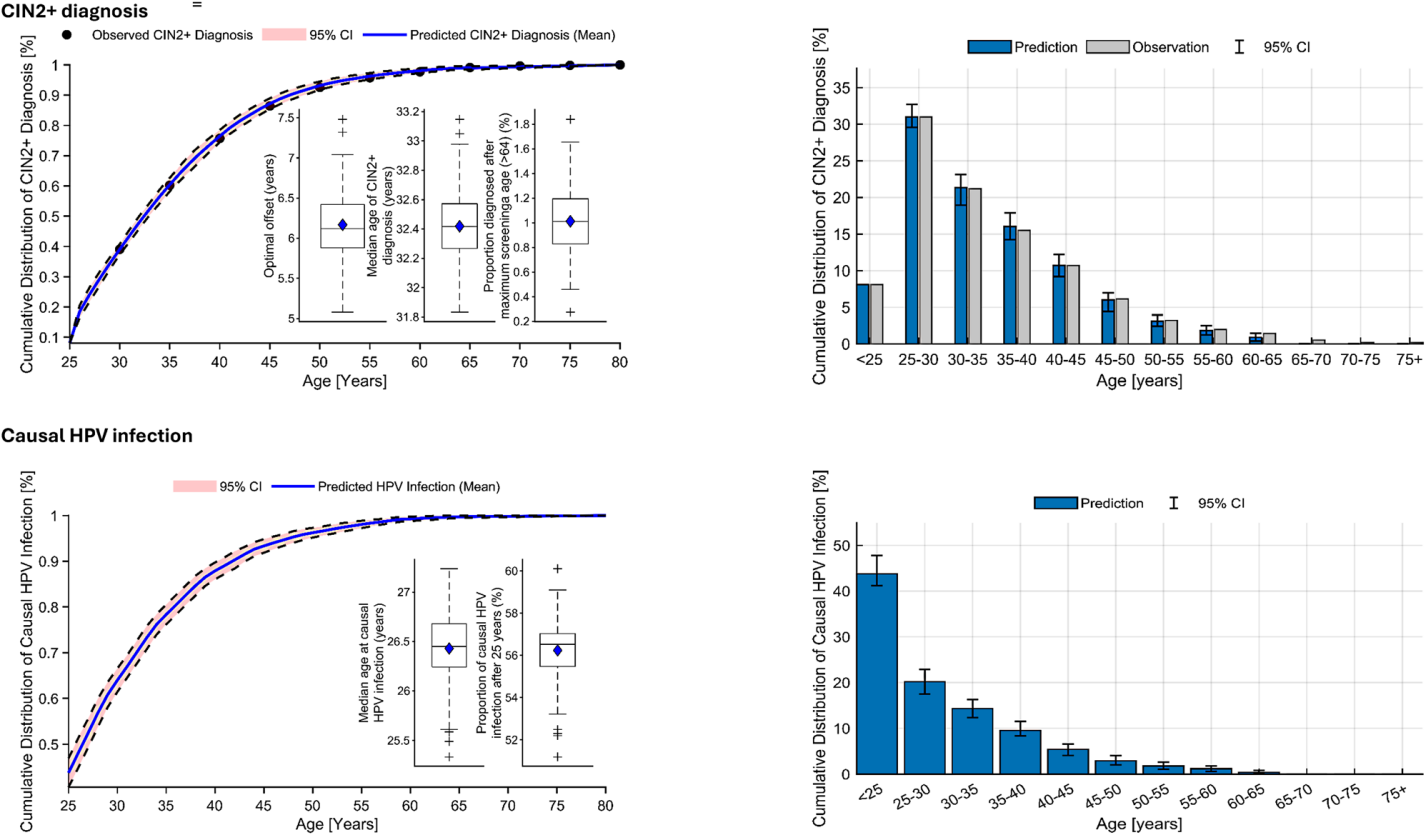
Cumulative age distributions of observed and predicted CIN2 + diagnosis and predicted HPV infection. CIN2+, cervical intraepithelial neoplasia grades 2, 2/3, 3, and adenocarcinoma; HPV, Human papillomavirus; CI, confidence interval

Table 1 presents the summary results of the base case analysis. The optimal time between causal HPV infection and CIN2 + diagnosis, i.e., the offset, was 6.17 (95% CI 6.08, 6.26) years. The median ages (95% CI) of causal HPV infection and disease diagnosis among women diagnosed with CIN2 + were estimated to be 26.43 (26.36, 26.50) years and 32.42 (32.37, 32.47) years, respectively. Over half (56.24%, 95% CI 55.93, 56.55) of causal HPV infections were estimated to occur after the age of 25 years. A small percentage (1.01, 95% CI 0.95, 1.07) of cases were estimated to be diagnosed after the age of 64 years (the maximum cervical cancer screening age). The cumulative time-to-event graphs, comparing the cumulative age distribution for predicted and observed CIN2 + diagnosis and predicted HPV infection, are presented in Fig. 2.

**Table 1:** Base case results

Parameter	Value (95% CI)
Optimal offset, years	6.17 (6.08, 6.26)
Median age at causal HPV infection, years	26.43 (26.36, 26.50)
Median age at CIN2 + diagnosis, years	32.42 (32.37, 32.47)
Percent causal HPV infections after age 25	56.24% (55.93, 56.55)
Percent causal HPV infections after age 35	21.65% (21.43, 21.87)
Percent causal HPV infections after age 45	6.65% (6.50, 6.80)
Percent diagnosed after maximum screening age (> 64 years)	1.01% (0.95, 1.07)

CI, confidence interval; CIN2+, cervical intraepithelial neoplasia grades 2, 2/3, 3, and adenocarcinoma; HPV, human papillomavirus

### Scenario analyses

Scenario analysis results are presented in Table 2. Across scenarios, as the percentage of censoring increases, the optimal offset increases. The offset increased from 5.82 (95% CI 5.48, 6.16) years in scenario 1 to 7.09 (95% CI 6.79, 7.39) years in scenario 4, and the median age of causal HPV infection decreased from 26.87 (95% CI 26.63, 27.11) years in scenario 1 to 25.59 (95% CI 25.25, 25.93) years in scenario 4. The median age of CIN2 + diagnosis remained relatively stable across scenarios 1–4 at approximately 32 years. The percentage of causal HPV infections that occurred after the age of 25 slightly decreased from scenario 1 (58.16%, 95% CI 56.95, 59.37) to scenario 4 (52.60%, 95% CI 51.10, 54.10). The proportion of diagnoses made after the maximum age of screening increased from 0.96% (95% CI 0.76, 1.16) in scenario 1 to 1.09% (95% CI 0.93, 1.25) in scenario 4. Significant differences were demonstrated between the scenarios for the optimal offset, median age of causal infection, and the proportion of causal HPV infections after age 25. Pairwise differences were not significant between scenarios except for the pairwise comparison of scenario 4 vs. scenario 2 (*P* = 0.0343). The cumulative time-to-event graphs, comparing the cumulative age distribution for predicted and observed CIN2 + diagnosis and predicted HPV infection, for scenarios 1–4 are presented in Supplemental Fig. 1 and boxplots of outcomes for scenarios 1–4 are presented in Supplemental Fig. 2.

**Table 2:** Scenario analysis results

Outcome	Scenario 1	Scenario 2	Scenario 3	Scenario 4	*P* value
Optimal offset, years	5.82 (5.48, 6.16)	6.05 (5.76, 6.34)	6.43 (6.17, 6.69)	7.09 (6.79, 7.39)	< 0.001
Median age at causal HPV infection, years	26.87 (26.63, 27.11)	26.65 (26.46, 26.84)	26.24 (26.01, 26.47)	25.59 (25.25, 25.93)	< 0.001
Median age at CIN2 + diagnosis, years	32.48 (32.42, 32.54)	32.48 (32.32, 32.64)	32.47 (32.29, 32.65)	32.54 (32.36, 32.72)	0.871
Causal HPV infections after age 25, %	58.16% (56.95, 59.37)	57.28% (56.36, 58.2)	55.76% (54.59, 56.93)	52.60% (51.10, 54.10)	< 0.001
Diagnosed after maximum screening age (> 64 years), %	0.96% (0.76, 1.16)	0.99% (0.77, 1.21)	1.03% (0.83, 1.23)	1.09% (0.93, 1.25)	0.712

CIN2+, cervical intraepithelial neoplasia grades 2, 2/3, 3, and adenocarcinoma; HPV, human papillomavirus

Data shown as value (95% CI)

Under each censoring assumption, we recalculated the cumulative percentage of total CIN2 + cases observed over the first 3 years and refitted the gamma distribution so that the model time from infection to onset (i.e., the offset) was varied one at a time around their base case values for the following scenarios using a gamma distribution: Scenario 1: 0% censoring + gamma (0.7, 1.1); Scenario 2: 5% censoring + gamma (0.5, 2.0); Scenario 3: 10% censoring + gamma (0.5, 2.75); and Scenario 4: 20% censoring + gamma (0.5, 4.0)

### Sensitivity analyses

In the sensitivity analysis where an alternate parametric exponential distribution with a lambda of 1.2 was used for simulating the time from causal infection to onset of CIN2 + rather than a gamma distribution, no statistically significant difference in outcomes were observed (Table 3). The exponential distribution vs. the gamma distribution exhibited a lower mean offset (5.88 [95% CI 5.58, 6.18] vs. 6.02 [95% CI 5.78, 6.26] years). Supplemental Fig. 3 depicts the time-to-event graphs, comparing the cumulative age distribution for predicted and observed CIN2 + diagnosis and predicted HPV infection, and boxplots for outcomes for the gamma and exponential distributions are presented in Supplemental Fig. 4.

**Table 3:** Gamma vs. exponential distribution sensitivity analysis results

Outcome	Gamma (95% CI)	Exponential (95% CI)	*P* value
Optimal offset, years	6.02 (5.78, 6.26)	5.88 (5.58, 6.18)	0.455
Median age at causal HPV infection, years	26.64 (26.46, 26.82)	26.82 (26.62, 27.02)	0.192
Median age at CIN2 + diagnosis, years	32.47 (32.39, 32.55)	32.46 (32.37, 32.55)	0.828
Causal HPV infections after age 25, %	57.41% (56.59, 58.23)	57.92% (56.87, 58.97)	0.468
Percent diagnosed after maximum screening age (> 64 years), %	0.97% (0.78, 1.16)	0.95% (0.79, 1.11)	0.887

CI, confidence interval; CIN2+, cervical intraepithelial neoplasia grades 2, 2/3, 3, and adenocarcinoma; HPV, human papillomavirus

In the sensitivity analysis that assumed the time from causal HPV infection to CIN2 + disease onset was age-dependent, with slower progression for infections acquired at older ages of onset, the fitting of the model to the observed data was slightly improved (Table 4). The median age for CIN2 + diagnosis analysed across varying age factors remained stable at 32 years. The proportion of diagnoses occurring after the maximum screening age for the corresponding age factors showed a small increase as age dependency increased. The median age for CIN2 + causal infection and the proportion of individuals acquiring infection after the age of 25 decreased as age factor increased (*P* < 0.001). The cumulative time-to-event graphs, comparing the cumulative age distribution for predicted and observed CIN2 + diagnosis and predicted HPV infection are presented in Supplemental Fig. 5 and boxplots of outcomes are presented in Supplemental Fig. 6. Alternatively, increasing age-related factors—whereby a later age of causal HPV acquisition leads to faster progresses to CIN2+—were associated with a statistically significant monotonic decrease in the optimal offset between HPV infection and CIN2 + diagnosis (from 6.02 years [95% CI 5.75, 6.29] to 4.97 years [95% CI 4.64, 5.29]; *P* < 0.001; Supplemental Table 2). Correspondingly, the median age at causal HPV infection increased from 26.64 years (95% CI 26.43, 26.85) to 27.73 years (95% CI 27.48, 27.97; *P* < 0.001), while the median age at CIN2 + diagnosis remained stable across scenarios (approximately 32.5 years; *P* = 0.85). The proportion of causal HPV infections occurring after age 25 rose significantly from 57.41% (95% CI 56.47, 58.35) to 62.10% (95% CI 60.49, 63.70; *P* < 0.001). In contrast, the proportion of CIN2 + diagnoses occurring after age 64 remained low and statistically unchanged (ranging from 0.88% to 0.97%; *P* = 0.40). The cumulative time-to-event graphs, comparing the cumulative age distribution for predicted and observed CIN2 + diagnosis and predicted HPV infection are presented in Supplemental Fig. 7 and boxplots of outcomes are presented in Supplemental Fig. 8.

**Table 4:** Age-dependency sensitivity analysis results, in which a later age of causal HPV acquisition progresses to CIN2 + more slowly

Outcome	Age factors					*P* value
	0	0.025	0.05	0.1	0.2	
Optimal offset, years	6.02 (5.75, 6.29)	6.15 (5.78, 6.52)	6.52 (6.23, 6.81)	6.70 (6.41, 6.99)	7.29 (6.83, 7.75)	< 0.001
Median age at causal HPV infection, years	26.64 (26.43, 26.85)	26.52 (26.25, 26.79)	26.31 (26.08, 26.54)	25.97 (25.77, 26.17)	25.25 (24.63, 25.87)	< 0.001
Median age at CIN2 + diagnosis, years	32.47 (32.38, 32.56)	32.53 (32.32, 32.74)	32.52 (32.38, 32.66)	32.48 (32.37, 32.59)	32.48 (32.34, 32.62)	0.952
Causal HPV infections after age 25, %	57.41% (56.47, 58.35)	56.97% (55.81, 58.13)	56.19% (54.95, 57.43)	54.85% (53.71, 55.99)	51.72% (49.01, 54.43)	< 0.001
Percent diagnosed after maximum screening age (> 64 years), %	0.97% (0.75, 1.19)	1.06% (0.91, 1.21)	1.13% (0.94, 1.32)	1.21% (1.04, 1.38)	1.22% (0.96, 1.48)	0.220

CI, confidence interval; CIN2+, cervical intraepithelial neoplasia grades 2, 2/3, 3, and adenocarcinoma; HPV, human papillomavirus

Data shown as value (95% CI)

## Discussion

Our model determined the age distribution of predicted HPV infection in England, revealing that a substantial proportion (56.24%) of causal HPV infection occurs after the age of 25 years, the age at which women are no longer eligible for reimbursed vaccination in England under the HPV NIP. The median age of diagnosis was 32 years, and some women were diagnosed after the maximum screening age. Results were robust to variations in inputs and parameterisation tested in scenario and sensitivity analyses. These findings suggest that expanding vaccination catch-up opportunities beyond the current eligible age within the NIP in England could avert a substantial burden of disease-causal HPV infection, high-grade cervical disease, and subsequent cervical cancer among women over the age of 25 years. The findings support the continued benefit of providing catch-up opportunities for missed vaccinations in schools. The roll out of a single dose HPV vaccination programme in the UK from September 2023 emphasises the importance of catch-up opportunities as the number of vaccination contact points in schools is reduced. Further research on the public health and economic impacts is needed to support implementation strategies for extending the age of eligibility. NHS England has pledged to eliminate cervical cancer by 2040, and a key pillar of this pledge is the improvement in the uptake of HPV vaccination and cervical screening [30].

Previous US modelling studies have evaluated similar outcomes to our study [25–27]. Using a compartmental disease model to examine the impact of catch-up HPV vaccination on disease and economic outcomes, Daniels et al. (2021) estimated the median age for HPV infection in the US in men and women at 25–26 years [25]. However, since they used a compartmental model, they were unable to estimate the age distribution of causal infection. Similar to our study, Prabhu et al. (2021) [26] and Burger et al. (2019) [27] conducted studies based on a microsimulation where females were followed over time, utilising data on the time from causal infection to subsequent health states, and considered the potential impact of cervical cancer screening to estimate age at causal infection based on US epidemiological data. Using data from a state-wide surveillance registry in Connecticut, Prabhu et al. estimated the mean age of causal HPV infections at 23.9 years and calculated that approximately 42.7% of causal infections occurred among women aged 27 years or older [26]. Using 4 Cancer Intervention and Surveillance Modeling Network (CISNET)-cervical models, Burger et al. [27] incorporated time from causal infection to high-grade precancer (i.e., CIN2 or CIN3), from high-grade precancer to invasive cancer onset, and from invasive cancer onset to cancer detection. In 3 of the models, in the absence of screening, the median age of acquisition of causal HPV infection was between ages 19–23 years, while the other model projected HPV infection to occur later at 34 years. The dwell time from acquisition to high-risk HPV infection to cancer detection was shortest among the model that projected the later age (17.5 years vs. 25–26 years with the other models) [27]. When imperfect compliance with US guidelines for screening, which mirrors the real world, was assumed in a sensitivity analysis, the median age of acquiring a causal high-risk HPV infection was projected at 25.1, 25.4, 27.9, and 49.9 years [27]. Our study’s findings are consistent with these 3 studies [25–27], showing that disease-causal HPV infection continues to occur among women aged > 25 years.

Our model simulates women who had a causal infection and progressed to CIN2+; therefore, mortality or clearance of infections were not incorporated into the model. Similar to Prabhu et al. [26], we used data on time from causal infection to onset of CIN2 + from the VIVIANE [24] and FUTURE I HPV vaccine clinical trials [5]. These clinical trial data use sensitive assays with regular measurements that allow for the adequate detection of HPV DNA and the determination of histologic endpoints for HPV types that are rigorously analysed by a panel of pathologists.

As with any microsimulation study, the findings should be interpreted in the context of the limitations and assumptions. By using screening to estimate the median age of women acquiring causal HPV infections, the model does not differentiate between lesions that result from newly acquired infections and lesions that result from HPV infections reactivating after a period of latency. As a result, the model cannot determine whether an infection is a new infection or one based on an earlier infection (at an unknown age) that was reactivated. The model also relies on screening to determine the age of diagnosis; therefore, the model cannot estimate cases of CIN2 + diagnosed with methods other than regular screening tests. Our model also does not distinguish between the different oncologic HPV types because the underlying CIN2 + data was not available. Lastly, the model considers detected CIN2 + cases only and the impact of vaccination is not incorporated into the model. Undetected or missed CIN2 + cases may progress to cervical cancer and could potentially be prevented through vaccination. Given the historically high rates of HPV vaccination in England, the median age of infection, CIN2+, and diagnosis would presumably be higher than it is in the current analysis.

Several assumptions in the model were made due to the lack of real-world data to inform certain inputs. The model assumes that the time from causal infection to CIN2 + onset does not differ by geography or age of causal infection. When we assumed that the time from causal infection to CIN2 + disease onset was age-dependent, with time increasing with older ages of onset in the sensitivity analysis, the fit of the model to the observed data was slightly improved. Also, the model assumes that the shape of the age distribution that caused HPV infection is similar to the observed age distribution of CIN2 + diagnosis. It is also important to note that, in 2003, the NHS modified its recommendation for the commencement of cervical cancer screening from age 20 to age 25 years; therefore, there is the possibility that some women who were screened at an earlier age were included in the current data. This might explain why approximately 8% of the CIN2 + diagnoses occurred before the earliest recorded age of screening (25 years of age). However, since the model requires screening to diagnose CIN2+, cases screened before the age of 25 years were excluded from the model calibration.

## Conclusions

This study estimates that a substantial proportion of causal HPV infections resulting in progression to high-grade CIN occur after the maximum age of eligibility for HPV vaccination as part of the reimbursed NIP in England, suggesting a potential benefit of vaccination beyond the age of 25 years. These findings support the need for both catch-up opportunities for those who missed vaccination in school, in addition to consideration of vaccination beyond the current programme’s age restriction.

## Appendix 1: Methods for age-dependency sensitivity analysis

In the base case, we assume the time from causal infection to CIN2 + onset does not depend on age. However, it is hypothesised that the time from causal infection to CIN2 + onset may increase with increasing age of causal infection. Therefore, we conducted a sensitivity analysis that determines the impact of age dependence in the time from causal infection to CIN2 + onset in estimating the median age of causal infection.

In the base case, it was assumed that the time from causal infection to CIN2 + onset follows a gamma distribution. Instead of applying the gamma(1,1) distribution uniformly across all ages, we specifically applied it to a single age. The gamma(1,1) distribution is utilised for females with a causal infection at 20 years of age, based on the age of females entering the FUTURE I study. To explore variation in age, we assumed that females with causal infections occurring earlier or later than 20 years of age exhibited different progression rates compared to those with causal infections at 20 years of age. This relationship can be described using the following equation:

- Time to CIN2 + onset = 0.5 years + gamma(a + b *age, 1),

where a is defined as 1–20*b to ensure that a gamma(1,1) distribution applies to a 20-year-old female, b ≥ 0, and age refers to the age at which the causal infection occurs. In the scenario where b = 0, we arrived at the base case, indicating that the time to CIN2 + onset is independent of the age at which the infection occurs.

In the base case, a constant was used to find the optimal offset between the age distributions for causal infection and CIN2 + diagnosis. In the sensitivity analysis, we assumed a linear relationship that depends on the age of causal infection: offset = C + D*Age(causal infection). The parameter C is constrained to ensure the age of causal infection is prior to the age of causal infection for each simulated female.

A grid search was performed in the model using a user-modifiable range of values for coefficients “c” and “D” (where c and D are non-negative) to find the optimal parameters for the offset (based on the chi-square goodness-of-fit statistics). Here, C = c - D*min(Age(diagnosis)) so that Age(causal infection) < = Age(diagnosis). The density of the grid is specified by the user-modifiable number of steps for the two parameters. In the base case, c varies from 0 to 10 and D varies from 0 to 0.5, with 25 steps for both parameters.

In the sensitivity analysis, a macro allows the user to run the model for 4 alternative values for b; default values are set for b = 0.025, 0.05, 0.10, and 0.20, representing an increasing influence of age on the time from causal infection to CIN2 + onset (in years). The resulting distributions for the default values for b are given as follows:

- b = 0.025: 0.5 + gamma (0.5 + 0.025*age, 1),
- b = 0.05: 0.5 + gamma (0.0 + 0.05*age, 1),
- b = 0.1: 0.5 + gamma (-1.0 + 0.1*age, 1), and.
- b = 0.2; 0.5 + gamma(-3.0 + 0.2*age, 1).

The distribution of time from causal infection to CIN2 + onset for females with an age of causal infection ranged from 20 to 45 is presented in panels A-D for 4 values for b. A second macro allows the user to run a 2-way sensitivity analysis, varying both b (across 4 values) and the age of causal infection for the gamma(1,1) distribution (across 3 values).

**Figure:**
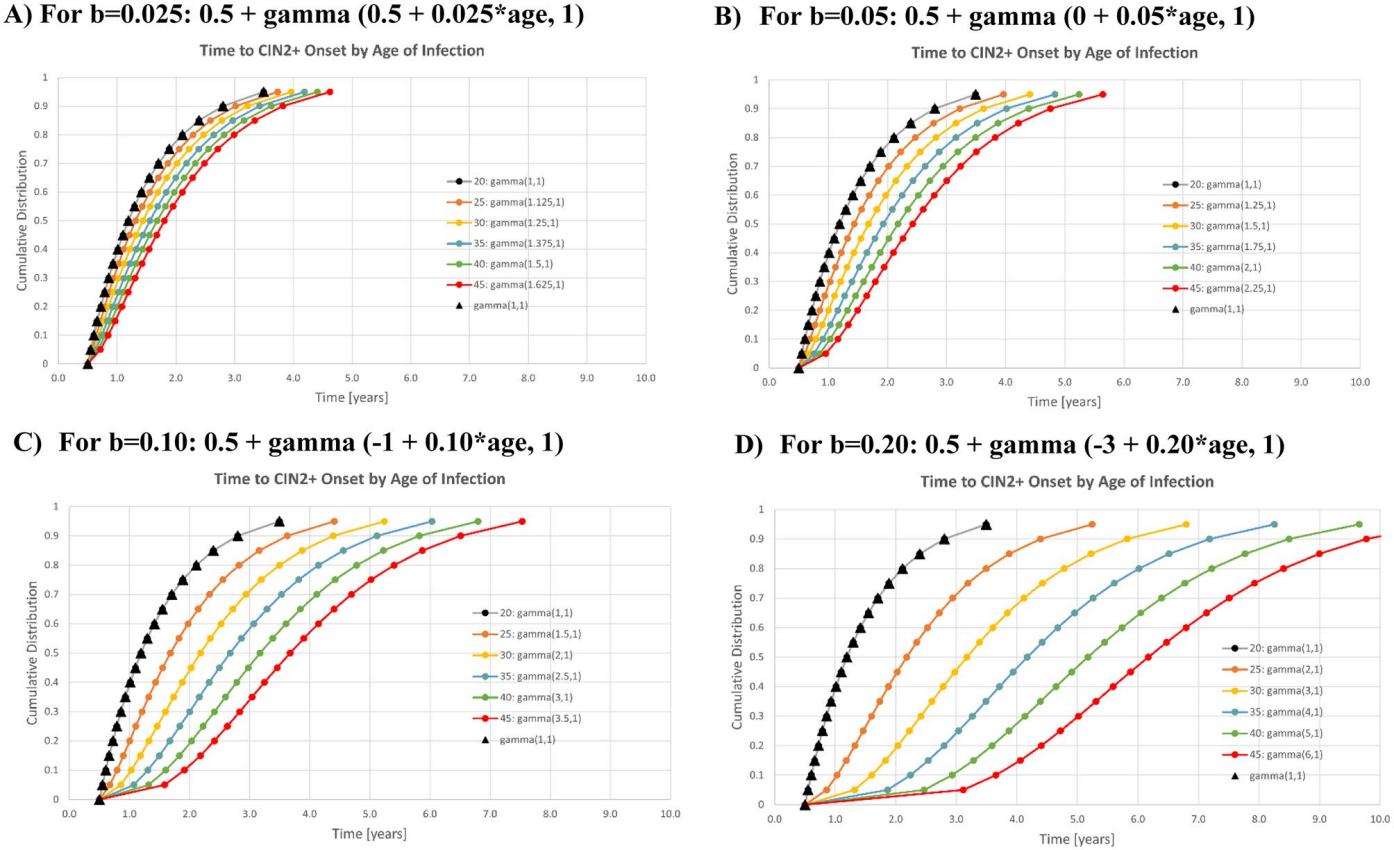

## Electronic supplementary material

Below is the link to the electronic supplementary material.

Supplementary Material 1

## Data Availability

Data sharing is not applicable to this article as no datasets were generated during the current study.

## Abbreviations

HPV: Human papillomavirus
CIN: Cervical intraepithelial neoplasia
CIN2+: Cervical intraepithelial neoplasia grades 2, 2/3, 3, and adenocarcinoma in situ
NIP: National Immunisation Programme
UK: United Kingdom
NHS CSP: National Health Service Cervical Screening Programme
US: United States
2vHPV: Bivalent human papillomavirus vaccine
4vHPV: Quadrivalent human papillomavirus vaccine
