# Supplementary material for: Age of acquisition of disease-causal human papillomavirus infection to high-grade cervical intraepithelial neoplasia (CIN2+) in England
Source: BMC Infect Dis. 2026 Mar 31;26:680. doi: 10.1186/s12879-026-12686-z (PMC13036974; doi:10.1186/s12879-026-12686-z)
Supplement: Supplementary file 1 — Supplementary Material 1 [file 12879_2026_12686_MOESM1_ESM.docx]

Supplemental Table 1. Summary of model inputs

| **Parameter** | **Input** |  |
| --- | --- | --- |
| **Number of CIN2+ cases in England (2007–2008) ^A, B^** | |  |
| **Age category, n (cumulative distribution)** | |  |
| 25–29 years | 12548 (33.7%) |  |
| 30–34 years | 8577 (56.7%) |  |
| 35–39 years | 6276 (73.6%) |  |
| 40–44 years | 4333 (85.2%) |  |
| 45–49 years | 2487 (91.9%) |  |
| 50–54 years | 1297 (95.4%) |  |
| 55–59 years | 792 (97.5%) |  |
| 60–64 years | 576 (99.1%) |  |
| 65–69 years | 210 (99.6%) |  |
| 70–74 years | 76 (99.8%) |  |
| 75+ years | 67 (100.0%) |  |
| **Annual screening rates in England (2007–2008) ^A, C^** | |  |
| **Age category** |  |  |
| 25–29 years | 25.5% |  |
| 30–34 years | 31.6% |  |
| 35–39 years | 34.7% |  |
| 40–44 years | 35.9% |  |
| 45–49 years | 36.4% |  |
| 50–54 years | 22.5% |  |
| 55–59 years | 20.1% |  |
| 60–64 years | 18.7% |  |
| **3-year screening rates in England (2006–2008) ^A^** | | |
| **Age category** | |  |
| 25–29 years | | 58.6% |
| 30–34 years | | 68.0% |
| 35–39 years | | 72.2% |
| 40–44 years | | 73.7% |
| 45–49 years | | 74.3% |
| 50–54 years | | 72.0% |
| 55–59 years | | 67.4% |
| 60–64 years | | 64.5% |

CIN2+, cervical intraepithelial neoplasia grades 2, 2/3, 3, and adenocarcinoma; NHS, National Health Service

^A^ Data source: NHS Cervical Screening Programme England (2007 –2008) [[29](#_ENREF_29)].

^B^ After truncating cases before the age of 25.

^C^ Calculated using following equation: Annual rate=1-{1-[3 year screening rate]}^(1/3).

**Supplemental Table 2. Age-dependency sensitivity analysis results, in which a later age of causal HPV acquisition progresses to CIN2+ more rapidly**

| **Outcome** | **Age factors** | | | | | ***P* value** |
| --- | --- | --- | --- | --- | --- | --- |
|  | **0** | **-0.025** | **-0.05** | **-0.1** | **-0.2** |  |
| Optimal offset, years | 6.02 (5.75, 6.29) | 5.84 (5.50, 6.18) | 5.64 (5.35, 5.93) | 5.25 (4.86, 5.63) | 4.97 (4.64, 5.29) | <0.001 |
| Median age at causal HPV infection, years | 26.64 (26.43, 26.85) | 26.86 (26.59, 27.13) | 27.04 (26.85, 27.24) | 27.44 (27.08, 27.80) | 27.73 (27.48, 27.97) | <0.001 |
| Median age at CIN2+ diagnosis, years | 32.47 (32.38, 32.56) | 32.49 (32.33, 32.65) | 32.49 (32.33, 32.65) | 32.48 (32.26, 32.70) | 32.54 (32.35, 32.72) | 0.85 |
| Causal HPV infections after age 25, % | 57.41% (56.47, 58.35) | 58.00% (56.86, 59.14) | 58.85% (57.85, 59.85) | 60.94% (59.12, 62.76) | 62.10% (60.49, 63.70) | <0.001 |
| Percent diagnosed after maximum screening age (>64 years), % | 0.97% (0.75, 1.19) | 0.88% (0.73, 1.04) | 0.88% (0.73, 1.04) | 0.91% (0.74, 1.08) | 0.94% (0.80, 1.08) | 0.40 |

CI, confidence interval; CIN2+, cervical intraepithelial neoplasia grades 2, 2/3, 3, and adenocarcinoma; HPV, human papillomavirus

Data shown as value (95% CI).

Supplemental Figure 1. Cumulative distribution of predicted age distribution for observed and predicted CIN2+ diagnosis and predicted HPV infection for scenarios 1–4

CI, confidence interval; CIN2+, cervical intraepithelial neoplasia grades 2, 2/3, 3, and adenocarcinoma; HPV, Human papillomavirus

Under each censoring assumption, we recalculated the cumulative percentage of total CIN2+ cases observed over the first 3 years and refitted the gamma distribution so that the model time from infection to onset (i.e., the offset) was varied one at a time around their base case values for the following scenarios using a gamma distribution: Scenario 1: 0% censoring + gamma (0.7, 1.1); Scenario 2: 5% censoring + gamma (0.5, 2.0); Scenario 3: 10% censoring + gamma (0.5, 2.75); and Scenario 4: 20% censoring + gamma (0.5, 4.0).

Supplemental Figure 2. Boxplots of outcomes for scenarios 1–4

CIN, cervical intraepithelial neoplasia; CIN2+, cervical intraepithelial neoplasia grades 2, 2/3, 3, and adenocarcinoma; HPV, Human papillomavirus

Under each censoring assumption, we recalculated the cumulative percentage of total CIN2+ cases observed over the first 3 years and refitted the gamma distribution so that the model time from infection to onset (i.e., the offset) was varied one at a time around their base case values for the following scenarios using a gamma distribution: Scenario 1: 0% censoring + gamma (0.7, 1.1); Scenario 2: 5% censoring + gamma (0.5, 2.0); Scenario 3: 10% censoring + gamma (0.5, 2.75); and Scenario 4: 20% censoring + gamma (0.5, 4.0).

Scenario 1 labelled as 0%; Scenario 2 labelled as 5%; Scenario 3 labelled as 10%; and Scenario 4 labelled as 20% on the x-axis in the figure.

Supplemental Figure 3. Cumulative distribution of predicted age distribution for observed and predicted CIN2+ diagnosis and predicted HPV infection for the gamma vs. exponential distribution sensitivity analysis

CIN2+, cervical intraepithelial neoplasia grades 2, 2/3, 3, and adenocarcinoma; HPV, Human papillomavirus

Supplemental Figure 4. Boxplots for outcomes of sensitivity analysis examining gamma vs. exponential distribution

CIN2+, cervical intraepithelial neoplasia grades 2, 2/3, 3, and adenocarcinoma; HPV, Human papillomavirus

Supplemental Figure 5. Cumulative distribution of predicted age distribution for observed and predicted CIN2+ diagnosis and predicted HPV infection for the age-dependency sensitivity analysis, in which a later age of causal HPV acquisition progresses to CIN2+ more slowly

CIN2+, cervical intraepithelial neoplasia grades 2, 2/3, 3, and adenocarcinoma; HPV, Human papillomavirus

Supplemental Figure 6. Boxplots for outcomes of sensitivity analysis examining age-dependency, in which a later age of causal HPV acquisition progresses to CIN2+ more slowly

CIN, cervical intraepithelial neoplasia; CIN2+, cervical intraepithelial neoplasia grades 2, 2/3, 3, and adenocarcinoma; HPV, Human papillomavirus

Supplemental Figure 7. Cumulative distribution of predicted age distribution for observed and predicted CIN2+ diagnosis and predicted HPV infection for the age-dependency sensitivity analysis, in which a later age of causal HPV acquisition progresses to CIN2+ more rapidly

CIN, cervical intraepithelial neoplasia; CIN2+, cervical intraepithelial neoplasia grades 2, 2/3, 3, and adenocarcinoma; HPV, Human papillomavirus

Supplemental Figure 8. Boxplots for outcomes of sensitivity analysis examining age-dependency, in which a later age of causal HPV acquisition progresses to CIN2+ more rapidly

CIN, cervical intraepithelial neoplasia; CIN2+, cervical intraepithelial neoplasia grades 2, 2/3, 3, and adenocarcinoma; HPV, Human papillomavirus
